# Clinical evaluation of communication brain computer interfaces in amyotrophic lateral sclerosis: a landscape analysis

**DOI:** 10.3389/fnhum.2026.1771146

**Published:** 2026-05-22

**Authors:** Shana R. Melby, Jaganth Nivas Asok Kumar, Erin R. Bigus, Spencer Kellis

**Affiliations:** 1 Blackrock Neurotech, Salt Lake City, UT, United States; 2 University of Southern California Keck School of Medicine, Los Angeles, CA, United States; 3 USC Neurorestoration Center, Los Angeles, CA, United States

**Keywords:** ALS, amyotrophic lateral sclerosis, BCI, brain computer Interface, cBCI, clinical outcome assessment, COA, communication BCI

## Abstract

**Introduction:**

Amyotrophic lateral sclerosis (ALS) is a progressive motor neuron disease that leads to severe motor impairment, including loss of communication ability, and ultimately death. Communication brain computer interfaces (cBCIs) have the potential to restore communication without reliance on motor function, thereby improving quality of life, independence, and palliative care. However, standardized methods to evaluate cBCI efficacy necessary for clinical implementation are not yet established.

**Methods:**

We conducted a systematic literature review, semi structured interviews with key opinion leaders (KOLs), and a clinical assessment review panel to (1) identify clinical outcome assessments (COAs) relevant to cBCIs in ALS, (2) obtain expert feedback, and (3) synthesize the current clinical and scientific landscape.

**Results:**

A total of 21 COAs were identified as potentially relevant and may serve as a foundation for cBCI specific measures. However, no existing COA was found to comprehensively capture the clinical benefit or functional impact of cBCIs in ALS.

**Discussion:**

Current COAs are insufficient to evaluate cBCIs in ALS, highlighting a critical gap. Development of cBCI specific outcome measures is needed to support clinical validation, regulatory evaluation, and adoption.

## 1 Introduction

Amyotrophic lateral sclerosis (ALS) is a motor neuron disease characterized by progressive degeneration of upper and lower motor neurons, resulting in severe atrophy, fasciculations, spasticity, and hyper-reflexivity of muscles throughout the entire body. Clinical presentation and progression vary drastically across individuals. However, in all cases, motor neuron death inevitably spreads across the body causing progressive disability, paralysis, and mortality (Hardiman et al., 2017; van Es et al., 2017). There are an estimated 25,000 individuals in the US currently living with ALS, with an increasing prevalence and incidence of ALS diagnoses reported worldwide over the last decade (Longinetti and Fang, 2019).

One of the most feared end points of ALS progression is a condition called “locked-in syndrome” (LIS), which is characterized by tetraplegia and bulbar palsy (Das et al., 2022; Bauer et al., 1979). Leading up to this end stage, communication abilities progressively decline, particularly with the onset of bulbar symptoms. In one longitudinal study, speech deteriorated to a level requiring augmentative and alternative communication (AAC) methods in approximately 60% of participants, with speech remaining adequate for an average of about 18 months following the first bulbar symptom, accompanied by declines in articulation rate and intelligibility (Makkonen et al., 2018). Although speech therapy and augmentative and alternative communication (AAC) devices can be used to assist with speech degradation for a short period of time, these interventions are typically only effective in earlier stages of decline and complete loss of communication occurs in almost all patients. Because of this pervasive and rapid communication decline, patients are advised to make extremely difficult advanced care and end-of-life decisions very early in their diagnosis (van Es et al., 2017), and the progression of the disease to this locked-in state eliminates the patient’s ability to alter any of these decisions or communicate any additional wishes as their situation evolves. The loss of autonomy due to inability to communicate is a consistent feature of end-stage ALS, impacting not only the ability to manage end-of-life decisions but also day-to-day caregiver reliance, social relationships, medical care, perceived mental and physical health, and myriad other aspects of life, leading to a severe degradation in quality of life (Leigh et al., 2003; Green et al., 2003).

Communication brain-computer interfaces (cBCIs), a subset of BCIs, are neural decoding devices that can be invasive or non-invasive and are designed to enable written and/or verbal communication for paralyzed patients. cBCIs offer a unique opportunity for completely movement-free communication for those with severe paralysis due to ALS or other causes.

Despite the clear clinical potential of cBCIs for patients with ALS and other forms of severe paralysis, none have been successfully clinically applied on a meaningfully large scale. Furthermore, the wide variability between systems and methodological approaches makes evaluation of effectiveness difficult. Specific cBCIs in the literature have enabled keyboard control (Jarosiewicz et al., 2015), enhancement of residual speech function (Rubin et al., 2022; Wilson et al., 2020; Stavisky et al., 2019; Wairagkar et al., 2025), and restoration of speech without residual function (Chaudhary et al., 2022; Holz et al., 2015). Electrodes used to read neural activity for cBCIs also vary in invasiveness and include implanted percutaneous and transcutaneous microelectrodes, electroencephalography (EEG), magnetoencephalography (MEG), and electrocorticography (ECoG) (Brumberg et al., 2010). As a result of this wide variability in decoding, output, application, and electrode design, development of generalizable evaluation metrics for cBCI efficacy has been difficult. There are currently no cBCI-specific clinical outcome assessments (COAs) that have been validated or widely accepted as standardized measures to assess the efficacy of cBCIs in the ALS or any other patient population.

This study conducted a landscape analysis of current approaches to communication efficacy assessment within the context of clinically evaluating a future cBCI in the ALS patient population. We conducted a systematic literature review to identify current approaches for BCI and AAC devices, as well as COAs that are used for communication more broadly. This broad approach was taken to capture relevant information about analogous patient populations and to identify surrogate COAs (i.e., outcome measures designed for other cohorts and/or other applications) that may lend themselves well to cBCI assessment in ALS patients. We also conducted an interview study in which we identified and interrogated key opinion leaders (KOLs) in relevant fields who then provided information, feedback, and advice related to ALS, communication, cBCI, and related topics. Finally, we conducted a review panel to discuss the merits and relevance of COAs and assessment approaches identified through the preceding literature review and interview processes. This triangulated approach provides an evidence-based snapshot of the current scientific landscape and actionable insights for developing future cBCI-specific outcome measures.

## 2 Materials and methods

### 2.1 Systematic literature review

In order to identify existing measures that may be used or adapted to assess the effectiveness of cBCIs, we conducted a systematic literature review based on the PRISMA, 2020 and COSMIN guidelines, which were developed for systematic reviews specific to outcome measure identification and evaluation (Mokkink et al., 2018; Prinsen et al., 2018; Terwee et al., 2018; Page et al., 2021). For article selection, we created a broad and robust search filter using the COSMIN methodology guidelines (Terwee et al., 2018). This search filter includes specific terms for the constructs of interest (e.g., BCI), the population of interest and/or relevant patient populations, measurement or outcome assessment review properties (e.g., validation studies), and exclusion terms (e.g., animal research).

We included ALS, spinal cord injury (SCI), stroke, tetraplegia, locked-in syndrome, neuromuscular disease, cerebrovascular accident, Guillan-Barre syndrome, hemiparesis, motor neuron disease, myasthenia gravis, muscular dystrophy, and multiple sclerosis (MS) as relevant patient population search terms. Relevant constructs and technologies included communication, BCI, brain-machine interface, AAC, speech, and communication assistive technology. A complete list of the search terms used is included in the Supplemental material.

Two study personnel used the described search filter to conduct independent, exhaustive searches of PubMed, Embase, Scopus, and Cochrane Library databases. With the results from each database, after removing duplicate entries, they conducted a preliminary abstract screen to identify potentially relevant articles. Articles that were included after this screen were those that (1) were topically relevant to communication disability, (2) had peer-reviewed or equivalent validation of results, (3) contained original data or meta-analysis of original data, and (4) aimed to evaluate one or more properties of a COA, develop a COA, or evaluate the interpretability of a COA of interest (Prinsen et al., 2018). Studies that only used a COA as an outcome measure, rather than evaluating the COA itself, and those for which the full-text article was not available, were excluded. No date restrictions were applied to the search results.

After the preliminary abstract screening, each reviewer completed a more in-depth review of relevant articles and extracted the associated outcome measure of interest. Then, each outcome measure was included or excluded based on whether it could be used, either in-whole or in-part, to evaluate some component of efficacy related to cBCI in ALS patients. This includes assessments aimed at measuring: activities of daily living (ADLs), assistive technology (AT) performance, BCI performance, caregivers, cognitive and neurological ability, communication, and patient satisfaction and quality of life (QOL). If the primary source for an identified COA was not identified in the review, for example, if a COA was identified as a part of a meta-analysis of outcome measures, then the primary article in which that COA was developed and/or validated was identified and included subsequent to the primary literature review.

Following the in-depth article screening and COA extraction, the final articles and selected COAs from the independent reviewers were merged. All references and COAs appearing in both sets of results were retained in the final results. If a reference or COA appeared in only one reviewer’s list, it was reviewed by both the independent reviewers and a third individual to determine whether it should or should not be included.

The final list of potentially relevant COAs for cBCI evaluation in ALS was then compiled with the primary source article and copies of the outcome measure itself. Identified COAs were presented to KOLs for feedback during their interviews. The merits and relevance of each COA was also discussed in depth with a review panel of experts from relevant fields (see *Clinical Outcomes Review Panel*).

This review was informed by PRISMA guidelines, however, it was not designed as a fully PRISMA-conforming systematic review, as the objective was to identify and synthesize communication-related COAs referenced within the literature rather than to evaluate the studies themselves.

### 2.2 Key opinion leader interviews

We conducted a series of semi-structured interviews of KOLs to gain practical insight into current, relevant assessment tools, necessary assumptions made by those assessment tools, and existing gaps that would not be addressed if such tools were to be implemented as evaluation metrics for cBCI in ALS. The interview structure was developed iteratively, with pre-determined questions informed by a preliminary unstructured literature review of qualitative KOL interviews and refined through expert input and feedback from the FDA prior to finalization. KOLs were selected through purposive sampling based on their leadership within the field, as well as their considerable experience and positive reputations within their respective communities. KOL fields of interest included medicine (i.e., physicians), physical or occupational therapy, speech language pathology, ALS research, assistive and AAC devices, BCI used as AAC devices or cBCIs, and communication in individuals with severe language deficit (agnostic to patient diagnosis). In order to be considered for recruitment, KOLs were required to (1) be educated and employed in one of the selected fields of interest, (2) have an employment history of at least 10 years in the selected field or a demonstratable record of equivalent achievement, (3) be willing to spend at least 2 h participating in an interview, and (4) read, speak, and understand English fluently. Individuals with conflicts of interest, financial or otherwise, or those who disapprove of BCI as a viable technology for individuals with ALS were excluded.

Interviews consisted of 55 pre-determined open-ended questions with opportunity for follow-up and elaboration on emergent topics as appropriate. All KOLs were given the FDA definition of a COA and instructed that for the purposes of the interview, “we are considering COAs that could be used to evaluate how well an intervention, like a brain-computer interface (BCI), improves communication for an individual with ALS.” The succeeding questions were grouped into the same assessment categories as the COAs extracted in the literature review: ADLs, AT performance, BCI performance, caregivers, cognitive and neurological ability, communication, and patient satisfaction and QOL. We included questions specific to each category and generalized questions regarding each category’s relevance to ALS, cBCI, etc. We also solicited feedback on our categorization of assessments and COAs that had been identified as part of the literature review.

All interviews were conducted by the same interviewer, and it was made clear to all prospective KOLs that the research being conducted was disconnected from any company interests or relationships. Interviews were audio and video recorded and anonymized transcripts were used for all analyses. All KOLs provided informed consent for participation prior to initiation of study procedures. All study procedures and interview scripts were approved by Alpha IRB (San Clemente, CA).

#### 2.2.1 Thematic analysis of interviews

KOL interview responses were analyzed through thematic analysis, following the implementation outlined by Naeem et al. (2023). We began by selecting relevant quotations from the transcript of each response. From these quotations, we extracted keywords-individual words or phrases that best represented the response. After reviewing quotations and keywords, we defined codes that reflect specific but recurring ideas expressed by KOLs. Each response was then coded with one or more relevant codes. We next listed and organized codes into groups based on commonalities. These commonalities were used to identify themes, which are words or phrases that capture key ideas expressed by KOLs across questions and/or identify the relationship between question and response.

We used the identified themes to summarize KOL perspectives pertaining to each category. To elicit focused feedback on categories and COAs within, we quantified the number of occurrences of each theme, pooling across all questions within a category. Importantly, we excluded themes emerging from lack of awareness or expertise, responses to simple yes/no questions, and criticisms of the survey itself. The remaining top three themes were used to summarize KOL perspectives in each COA category.

The final coding and analysis of all interview transcripts were conducted by a single analyst. To enhance the rigor and consistency of the qualitative analysis, a second analyst independently coded a subset of the transcripts. This subsampling approach was used to calibrate the coding framework and ensure alignment in code application. Any discrepancies identified between analysts were discussed and resolved through consensus, thereby supporting the reliability and reproducibility of the coding process.

### 2.3 Clinical outcomes review panel

We assembled a panel of seven individuals through purposive sampling for an in-depth discussion on each COA identified as either part of the preceding literature review or KOL interviews. Panel members included a neuroscientist with experience in BCI research, a regulatory and clinical affairs specialist with experience in the medical device space, an industry product manager over medical devices, a business group leader responsible for the overall development and commercialization of cBCI within their company, two biomedical engineers with neurophysiology, BCI, neuroscience, and neurorehabilitation expertise, and a neuro-specialized occupational therapist. Members of the panel included the authors of this manuscript. However, there was no overlap between the KOLs interviewed and the panel members.

For each COA, the primary scientific article and a copy of the assessment were distributed to the panel for prior review. During the panel discussion, we provided members with a brief, objective summary of each COA, including its assessment category, target patient population (if specified), reporting type (e.g., patient reported outcome) and primary purpose. We then presented each scale, metric, etc., individually for open group discussion. The review panel meeting was audio and video recorded, and a transcript was used for analyses.

## 3 Results

### 3.1 Systematic literature review

**Figure 1:**
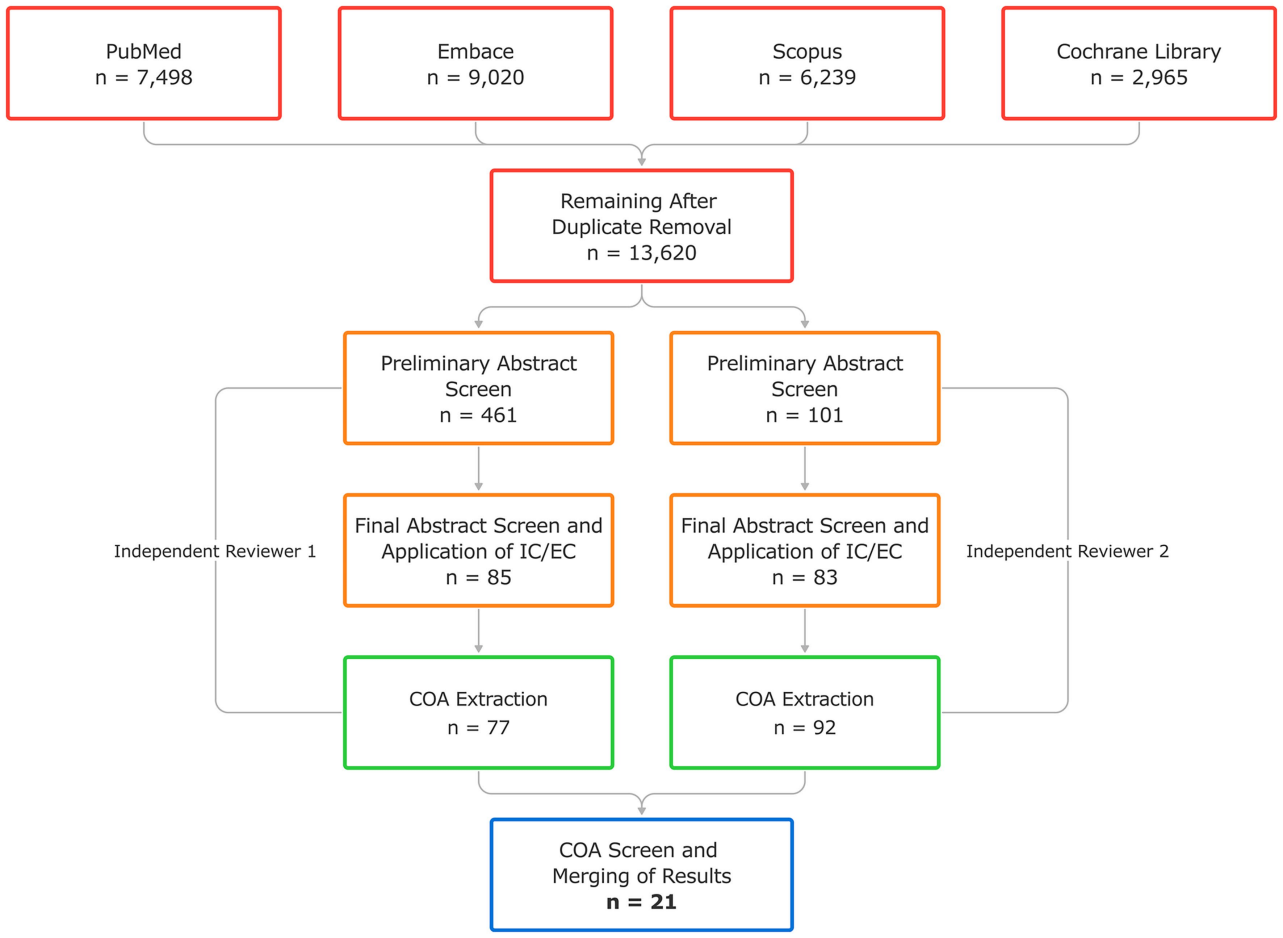
Flow chart of comprehensive literature review processes and results after merging independent review results. Initial database query and collation of abstracts [red] was completed using a single search filter, then abstract screening [orange], application of inclusion criteria (IC) and exclusion criteria (EC) [orange], and extraction of clinical outcome assessments (COAs) from remaining papers [green] was completed by two independent reviewers. Finally, the extracted COAs were screened and the results of the two independent reviews were merged to generate the final list of 21 COAs identified in the literature review process [blue].

In a systematic literature review based on a comprehensive search filter developed under COSMIN methodology guidelines (Terwee et al., 2018), we identified 13,620 unique articles across PubMed, Embase, Scopus, and Cochrane Library that met search criteria. Two individuals conducted independent reviews of these articles. After a preliminary abstract screening, 461 and 101 articles were identified by each reviewer as potentially relevant, warranting a more comprehensive review. After a secondary screen and application of inclusion criteria, each reviewer identified 85 and 83 papers, respectively, from which relevant COAs might be extracted. The first reviewer extracted 77 COAs from their identified 85 articles. The second reviewer extracted 92 COAs. After secondary review and merging the final list of COAs for each independent review, 21 were identified as potentially relevant in the context of cBCI in the ALS population. The literature search concluded in December 2023. Figure 1 shows a flow chart of the independent review processes and the results after merging. All resultant COAs and their associated articles are shown in Table 1. These COAs were presented to KOLs and the clinical outcomes review panel for discussion.

**Table 1:** COAs with relevance to the evaluation of cBCI in ALS.

COA	Summary	Literature review source(s)	Primary source (if different)
ALS Specific Quality of Life Instrument (ALSSQoL)	Patient reported outcome of QOL. Patients rate various items related to QOL. Scales include ratings of how large a problem has been, agreement with statements regarding their lives and disabilities, frequency of scenarios, etc.	Raheja et al. (2016)	Simmons et al. (2006)
American Speech Language Hearing Association’s Quality of Communication Life Scale (ASHA-QCLS)	Patient reported outcome of QOL and communication efficacy. Patients rate their agreement with statements regarding their perception of their quality of communication. Intended to be used in conjunction with other measures of cognition and functional communication.	Bose et al. (2009) and Rangamani and Judovsky (2020)	
Aphasia Communication Outcome Measure (ACOM)	Patient reported outcome of communication efficacy. Patients rate how effectively they can complete several communication tasks using a visual analog scale.	Lien et al. (2023)	Hula et al. (2015)
Care-Related Quality of Life Scale (CarerQoL)	Caregiver reported assessment. Caregivers select appropriate descriptions to fit their current caregiving situation (e.g., “I have [no, some, a lot of] fulfillment with carrying out my care tasks.”) and rate their current level of happiness using a visual analog scale.	Christie et al. (2022)	Brouwer et al. (2006)
Characters Per Minute	Quantitative performance metric. Measure of the number of characters per minute output for a patient using a device for digital communication.	Speier et al. (2013)	***
Classification Accuracy	Quantitative performance metric. Measure of the accuracy of a predictive classifier (i.e., how often a BCI outputs the correct word or words intended by the user)	McCane et al. (2014)	***
Communication Effectiveness Index (CETI)	Observer reported outcome of patient communication ability. Visual analog scale is used to rate a patient’s communication efficacy in a series of scenarios.	Charalambous et al. (2022)	Lomas et al. (1989)
Communication Outcome After Stroke (COAST)	Patient reported outcome of communication efficacy. Patients respond to a series of questions about their participation in various communication scenarios as well as the perceived impact of their communication on their QOL.	Doedens and Meteyard (2020)	Long et al. (2008)
Communicative Activity Log (CAL)	Observer reported outcome of patient communication ability. Patient ability is rated in various hypothetical scenarios. Includes both quality of communication and amount of communication sections (e.g., “how well would the patient communicate in scenario a? and, “how frequently does the patient communicate in scenario b?”).	Doedens and Meteyard (2020)	Pulvermüller and Berthier (2008)
Communicative Participation Item Bank (CPIB)	Patient reported outcome of communication efficacy. Patients rate how much their communication interferes with several communication tasks.	Sixt Börjesson et al. (2020)	Baylor et al. (2013)
Mutual Information	Quantitative performance metric. Uses prior information and computational modeling of errors to estimate accuracy and rate of information transmission.	Speier et al. (2013)	***
NASA-TLX	Patient reported outcome measure of cognitive load. Patients use a visual analog scale to rate the perceived mental demand, physical demand, temporal demand, performance, effort, and frustration level associated with a task.	Kübler et al. (2014)	Hart and Staveland (1988)
Perceived Stress Scale (PSS)	Caregiver reported assessment. Caregiver rates how often they experience various feelings, emotions, difficulties, etc. Secondary measure for caregiver burden.	Christie et al. (2022)	Lee (2012)
Practical Bit Rate	Quantitative performance metric. Measure of the speed of information transfer from a BCI to computer for speech output.	Speier et al. (2013)	***
Quebec User Evaluation of Satisfaction (QUEST 2.0)	Patient reported outcome measure of assistive technology efficacy. Patients rate their satisfaction with an assistive technology device.	Kübler et al. (2014)	Demers et al. (1996)
Short Sense of Competence Questionnaire (SSCQ)	Caregiver reported assessment. Caregivers rate their agreement with various statements about their interactions and perceptions of situations with the patient for whom they care.	Christie et al. (2022)	Jansen et al. (2007)
Speech and Pause Analysis (SPA)	Quantitative performance metric. Measure of the rate of speech for given statements. Patients are asked to read and repeat given statements. Primary output metrics include speaking rate (words per minute), total time duration, speech duration, pause duration, number of pause events, mean duration of pause and continuous speech events, and variation of pause and continuous speech events.	Barnett et al. (2020)	
The Speech Questionnaire	Observer reported outcome of patient communication ability. Patient ability and understanding is rated in various scenarios.	Lincoln (1982)	
The Voice Handicap Index-10 (VHI-10 lb)	Patient reported outcome of QOL and communication efficacy. Patients rate the frequency with which they experience various scenarios related to communication. Includes functional, physical, and emotional components to quantify patients’ perception of their disability related to vocal function.	Summaka et al. (2021)	Rosen et al. (2004)
Therapy Outcome Measure (TOM)	Clinician reported outcome of patient communication ability. Comprised of a series of open-ended questions, including probing follow up questions. Patient is rated on the quality of their response.	Hesketh et al. (2008)	
Word Symbol Rate	Quantitative Performance Metric. Measure of the maximum rate of symbol output by a BCI, scaled down by the error rate.	Speier et al. (2013)	

ALS, amyotrophic lateral sclerosis; BCI, brain computer interface; cBCI, communication brain computer interface; COA, clinical outcome assessment; QOL, quality of life. ***Primary sources for hardware and software performance metrics have not been included. These are used across a variety of contexts, most often for non-clinical purposes. Instead, we have included the literature review sources, which are, to our knowledge, the primary sources in which these metrics are used in the context of BCI or cBCI evaluation.

### 3.2 Key opinion leader interviews

To contextualize the findings of the literature review, we conducted semi-structured interviews with 15 KOLs across medical, research, and communication domains to gather perspectives on the evaluation of cBCI efficacy and the applicability of identified COA categories. Thematic analysis of the transcribed interviews was used to identify key insights regarding the validity, importance, and potential gaps in existing COAs.

**Figure 2:**
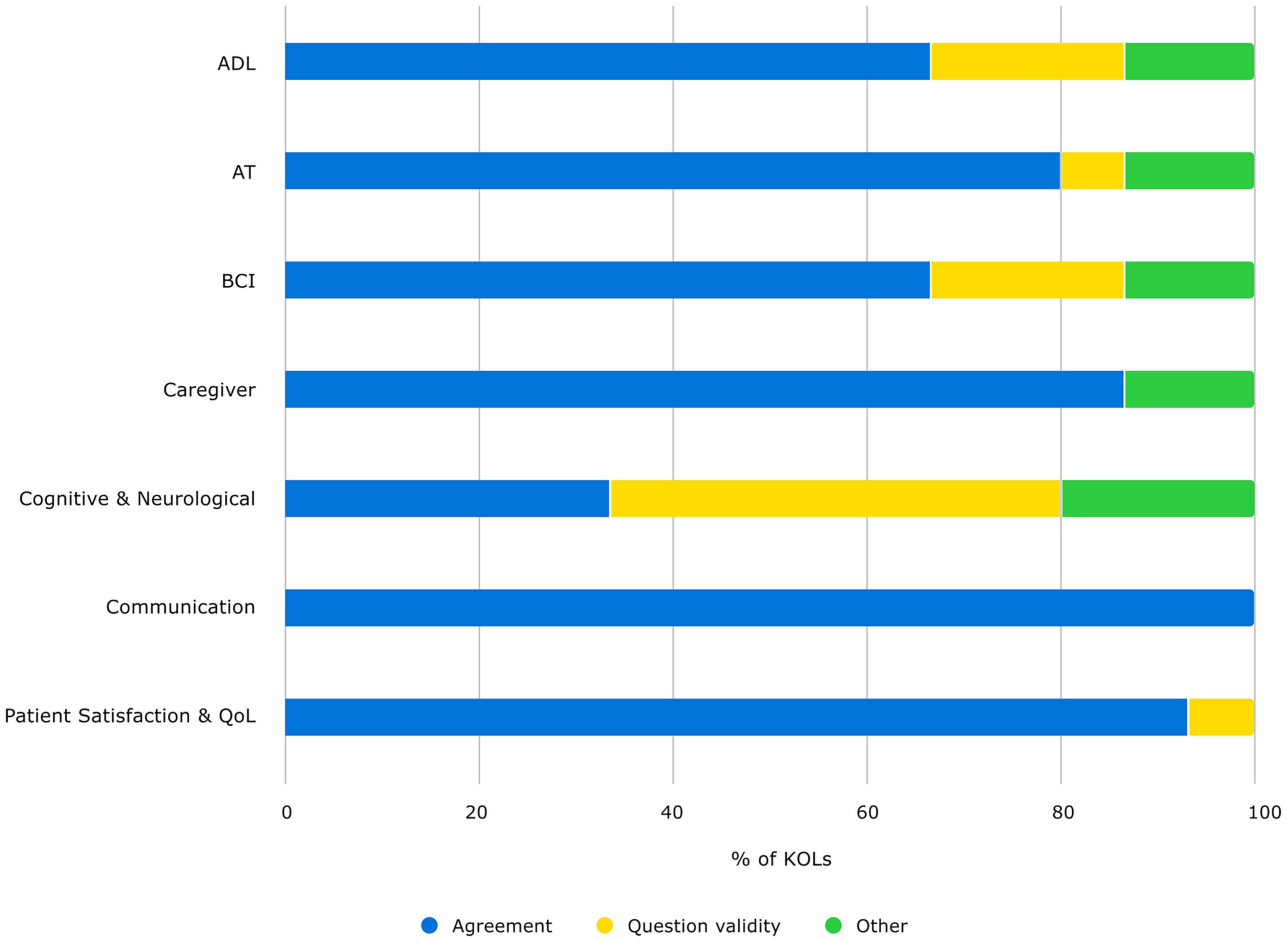
KOL feedback on category validity. Percent of KOLs who agree category is valid (blue), question validity (yellow), or express other ideas (green).

In general, the KOLs agreed on the validity of six out of the seven COA categories for evaluating cBCIs in ALS patient population, though some expressed concern about the complexity of measurements within different categories and the need for refinement to enable more accurate and precise evaluation within a cBCI context. Figure 2 shows a summary of KOL responses regarding the validity of each of the COA categories, where “Agreement” indicates that they considered the category valid, “Question Validity” indicates uncertainty or opposition to the category, and “Other” reflects a more complex response captured by other extracted themes.

**Figure 3:**
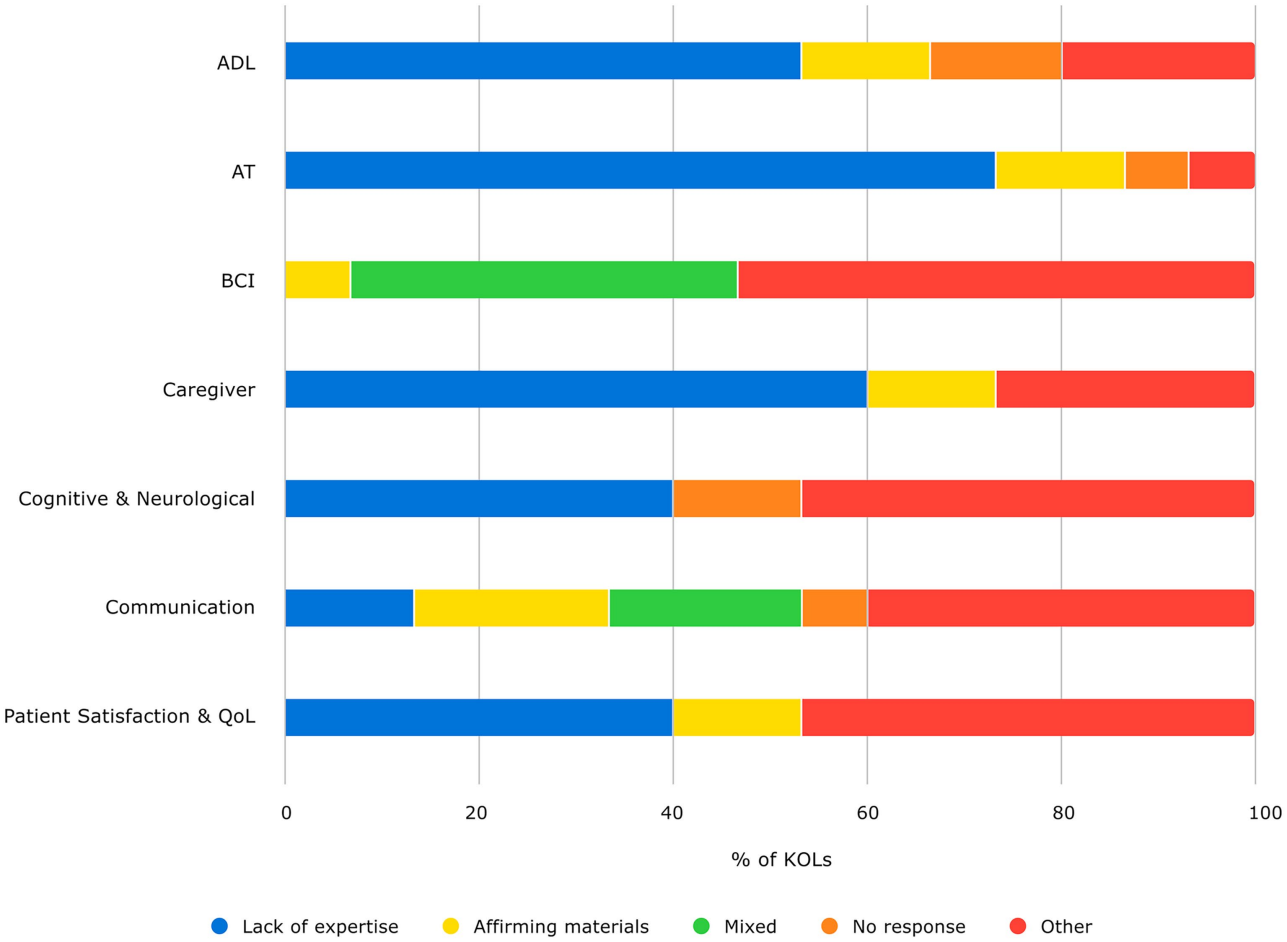
KOL feedback on COAs. Percent of KOLs who lack expertise with (blue), agree with (yellow), provide opinions in addition to agreement or lack of expertise (green), did not respond (orange), or express other opinions on (red) the provided list of COAs in each category.

From our thematic analysis, we identified 67 recurring codes and 32 themes across all KOL interviews. Table 2 shows all the extracted themes and examples of corresponding codes from the overall thematic analysis. To better understand the context of the response themes, we identified the top three themes in each COA category, shown in Figure 3. KOLs unanimously agreed on the importance of measuring communication, generally agreeing that communication effectiveness measures do not necessarily need to be specific to cBCI vs. a more generic assessment of communication ability. They also emphasized the importance of both asking patients about their communication goals and measuring communication ability quantitatively. The majority of KOLs supported measuring ADL performance, but many raised concerns that not all ADLs are relevant to cBCI, citing the need to consider the patient population, complexity of communication, and the need to use additional metrics to evaluate communication device efficacy. KOLs also supported both AT and BCI categories for evaluation of cBCIs in ALS populations. They highlighted the importance of measuring patients’ abilities to accurately communicate across contexts with and without the device and noted similarities between AT and BCI evaluation in this context. To meet the goal of evaluating communication devices, KOLs specified that device performance should be measured as it relates to communication, while also noting that other COA approaches from other categories should be included in a comprehensive assessment battery.

**Table 2:** Extracted themes from KOL interviews with associated codes.

Theme	Theme definition	Examples of relevant codes
Expand Communication Scope	Expand the scenarios in which a patient can communicate (in what situations can a patient communicate)	Self-sufficiency, mobility, usage, context breadth, vocabulary breadth, communication context
Measure Communication Scope	Measure extent of communication ability across scenarios (in what situations can a patient communicate)	Self-sufficiency, mobility, usage, context breadth, vocabulary breadth, communication context
Enhance Communication Quality	Improve qualities of communication (speed, accuracy, reliability) (is the communication understandable)	Accuracy, efficiency, reliability, failure modes
Measure Communication Quality	Measure qualities of communication (speed, accuracy, reliability) (is the communication understandable)	Accuracy, efficiency, reliability, failure modes
Monitor Wellbeing	Monitor patient physical and/or mental wellness	Quality of life, health, other diagnoses, disease progression, patient assessment
Improve Wellbeing	Improve patient physical and/or mental wellness	Quality of life, health, other diagnoses, disease progression, patient assessment
Gather Patient Perspective	Seek patient perspective/goals/feedback	Patient satisfaction, personalized
Individualized Approach	Create a plan based on an individual patient’s abilities/goals	Personalized
Patient Assessment	Evaluate physical or mental health/abilities	Patient limits, clinical tools, quantitative measures, qualitative measures, assess needs, patient report, patient assessment, support system report
Consider Patient Motivation	Evaluate willingness of patient to engage/participate	Patient limits, participation, patient assessment
Caregiver Demands	Abilities/limits/burden imposed on caregivers	Support system report, support system involvement, communication partner limits, caregiver burden, caregiving
Survey Caregiver Opinion	Request/use caregiver opinion of patient or system	Support system report, caregiving
Account for Declining Health	Consider the disease trajectory/changes in abilities	Early intervention, disease progression, patient limits
Barriers to Establishing Technology	Challenges or concerns that stand in the way of in someone choosing to get a BCI and/or making BCI widely available	Physical burden, barriers to widespread use, unique demands, failure modes, assessment challenges, eminence
Barriers to Daily Use	Once someone has a BCI, challenges that limit their desire or ability to use the device	Device burden, mental burden, communication partner limits, communication context
Account for Complexity	Topic or question is multifaceted and/or difficult to address	Complex, multifaceted, assessment challenges
Within Subject Controls	Collect and compare data taken from the same subject at different timepoints	Baseline measure, controlled comparison
Across Subject Controls	Collect and compare data taken across different subjects	Defined criteria, controlled comparison
Question Validity	Expresses doubt or uncertainty about relevance of prompt/proposed materials	Doubt, uncertainty, redundance, irrelevance
Question Feasibility	Agrees topic of interest is relevant but raises concerns about ability to execute/measure	Assessment challenges, doubt, uncertainty
Lack of Expertise	Does not weigh in on prompt due to lack of expertise	Unfamiliar
Unaware	When asked if aware of other tools/metrics, does not cite additional (but does not mention lack of expertise)	Unaware
Agreement	Agrees with prompt/validity	Affirmation
Affirming Materials	Agrees that prompt/proposed materials are adequate	Affirmation, affirming list, significant
Missing Factors	Notes additions that should be made to prompted materials	Rectify omission, not in isolation, secondary metric, suboptimal
Specify Parameters	Prompt/response is valid for certain conditions/applications	Conditional, disease specific, application specific, partial affirmation, increase specificity
Create Distinctions	Items in prompt/response should be studied distinctly	Draw distinctions
Unfulfilled Need	Points to an unmet need that warrants further work (e.g., a tool that should be developed)	Warrants further work
Parallels Between Disciplines	Assess multiple proposed measures similarly	Equivalence, unchanging
Technology Function	Reference to or expertise in technology (like BCI) performance	Device performance
Behavior	Reference to or expertise in daily behavior (including communication)	Motivated behavior
Neurobiology	Reference to or expertise in neurological underpinnings (of disease or behavior)	Neurological basis

BCI, brain computer interface.

Opinions were less consistent regarding cognitive and neurological, caregiver, and patient satisfaction and QOL COA categories. Few KOLs considered cognitive and neurological COAs as valid for assessing cBCI efficacy. They did, however, acknowledge that cognitive abilities impact device usage and that cognitive abilities are clinically measurable through both formal and informal conversation-based assessments. Additionally, KOLs advised that caregiver COAs as well as patient satisfaction and QOL COAs should be used as secondary or indirect measures of cBCI clinical benefit, and many indicated that they should only be measured specifically in terms of satisfaction with communication.

**Figure 4:**
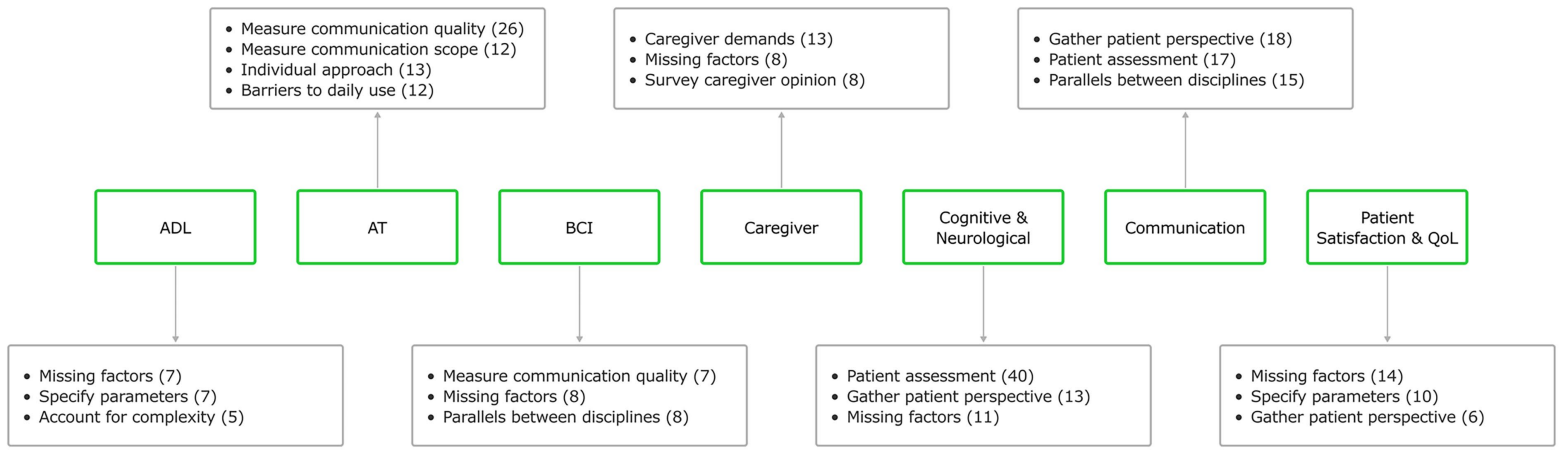
Key themes from KOL interview responses. The three most common themes (with number of occurrences), identified via systematic thematic analysis are shown for each of seven COA categories. Numbers are not normalized to account for different numbers of interview questions across categories.

Finally, we asked KOLs about specific COAs, whether identified in our literature review, or used by the KOL in their work with ALS or relevant patient populations. KOLs mentioned several COAs that they considered at least somewhat relevant to a cBCI and/or ALS context, some of which overlapped with COAs identified during the literature review. Those COAs mentioned by KOLs that were not identified during the literature review are shown in Table 3 along with their primary source article. After further discussion with KOLs and investigation into the COAs by the literature review team, none of the KOL-identified COAs were added to the final list of relevant COAs for cBCI in ALS. They were deemed irrelevant to this context or applicable only as screening tools for patient eligibility, rather than for evaluation of cBCI efficacy. Further, none of the KOLs indicated that they knew of any existing COA that could be used to accurately assess cBCI in ALS without some adaptation or supplemental evaluation. Many noted the need for the development of additional measurements as well as the need for validation in the ALS population specifically. Figure 4 shows the response profiles for the KOLs when shown the list of COAs identified from the literature review in each category, in which “Affirming Materials” indicates affirmation that the identified COAs were complete as far as the KOL’s knowledge regarding existing COAs in that category; “Mixed Response” indicates that they supplemented their response with feedback on specific COAs; “Other” indicates that they elaborated in some way on a lack of knowledge, affirmation of the materials (i.e., they provided more information or opinion than a simple affirmation or lack of expertise), or discussed additional opinions about existing COAs for that category; and “Lack of Expertise” indicates that the KOL did not feel they had sufficient knowledge in the category to provide feedback on existing COAs.

**Table 3:** COAs mentioned in KOL interviews not discovered during literature review.

COA	Number of KOLs	Type of assessment	Primary source article
Assistive Technology Device Predisposition Assessment	1	AT	Scherer and Cushman (2001)
Attention Network Test	1	Cognitive and Neurological	Fan et al. (2002)
Edinburgh Cognitive and Behavioral ALS Screen (ECAS)	5	Cognitive and Neurological	Abrahams et al. (2014)
False Positive/False Detection Rate	2	BCI Performance	–
Flanker Test	1	Cognitive and Neurological	Eriksen and Eriksen (1974)
Matching Person and Technology (MPT) assessment process	2	AT	Scherer and Craddock (2002)
Mini Mental State Examination (MMSE)	2	Cognitive and Neurological	Kurlowicz and Wallace (1999)
Montreal Cognitive Assessment (MoCA)	2	Cognitive and Neurological	Nasreddine et al. (2005)
Peabody Picture Vocabulary Test	1	Communication	Dunn and Dunn (1965)
Psychosocial Impact of Assistive Devices Scale	3	AT and ADL	Jutai and Day (2002)
Revised Amyotrophic Lateral Sclerosis Functional Rating Scale (ALSFRS-R)	1	Cognitive and Neurological	Maier et al. (2022)
Subjective Portability/Comfort	2	Patient Satisfaction and QOL	–
Classifier Vocabulary Size	1	BCI Performance	–

COA, cognitive outcome assessment; BCI, brain computer interface; KOL, key opinion leader; AT, Assistive Technology; ADL, Activities of Daily Living; QOL, Quality of Life. –Some assessments mentioned were not standard COAs, but conceptual considerations for evaluation. Thus, there is not an associated primary source article for these metrics.

### 3.3 Clinical outcomes review panel

We conducted a focused review panel in which a small group of experts discussed COAs identified from the literature review (see Table 1) and supporting literature to form a consensus on their applicability to cBCI evaluation in the ALS patient population. The panel discussed how each COA might be used either independently or as part of a battery of assessment tools that could be used to assess cBCI efficacy. The relevance of individual items within each COA, the merits of the scale types used, and the applicability of the questions/metrics to cBCI and/or the ALS patient population were also discussed. Within each category of assessments, the potential for the development of an assessment using an assortment of items from multiple COAs was examined. Performance metrics consisting of a single, quantitative measure (e.g., characters per minute), were discussed as a group.

Several themes emerged from panel discussions. First, the panel members uniformly agreed that the granularity of the Likert scale used for assessment was insufficient for the cBCI application. Second, performance metrics consisting of a single, quantitative measure (e.g., characters per minute), were deemed best suited to research rather than clinical assessment. The panel ultimately concluded that, although there are metrics that may be useful in the assessment of cBCI in ALS, there was not a single identified COA that could be used without some modification and none were comprehensive enough to be used alone. Discussion points and elaborations on thoughts and feedback from the review panel are included in the Discussion section.

## 4 Discussion

This study conducted a comprehensive systematic literature review and collected a series of expert opinions to evaluate the scientific landscape surrounding the assessment of communication, assistive technology, BCI, and the potential for the development of meaningful assessment approaches for cBCI in the ALS population. While several COAs were identified as a potential basis for a novel COA, none comprehensively or accurately captured the clinical benefit or functional impact of a cBCI for an ALS patient. Existing COAs do not fully account for key aspects of the ALS clinical context including sensitivity to disease progression, variability in functional communication abilities and corresponding cBCI use cases, and caregiver burden and thus cannot be used to evaluate cBCIs without modification.

### 4.1 Variability in communication deficits

A clear theme in the KOL interviews was the inability of existing COAs to address all forms of cBCI and all applications of cBCI to different stages of ALS. Deficits in communication vary substantially across individuals with an ALS diagnosis both because of the progressive nature of the condition (Brotman et al., 2024) and because of the unique expression of the disease in each individual. Within the average life expectancy of 20–48 months from onset (Chio et al., 2009), individuals decline from able-bodied toward LIS and eventual morbidity. The trajectory toward LIS varies substantially between individuals, with different functions including communication declining at different rates and with differing symptom profiles (Couratier et al., 2021; Din Abdul Jabbar et al., 2024). Cognitive deficits are often seen as worsening with disease progression (Crockford et al., 2018; Belkacem et al., 2020), although some studies have reported more stable cognitive performance over time (Finsel et al., 2023).

The variability and rapidity of changes in ALS progression presents a clear challenge to cBCI development and to the design of COAs. The sum of possible language deficits associated with ALS spans a broad range of cognitive and behavioral impairments (Pinto-Grau et al., 2021; Solca et al., 2023). Between 30 and 75% of ALS patients experience some form of cognitive decline whether from language, executive/attention, or other domains (Rusina et al., 2021), with up to 45% of patients meeting the criteria for frontotemporal dementia (FTD) diagnosis (Jellinger, 2023). Specific language dysfunction can include word naming, orthographic processing, and syntactic and grammatical processing (Pinto-Grau et al., 2021; Solca et al., 2023), in addition to dysarthria (Tomik et al., 2010). Given these characteristics, it would be challenging if not impossible for a single cBCI system to satisfy the needs of all individuals with ALS and all stages of the disease.

### 4.2 Diversity in cBCI approaches

Although none of the COAs were fit for purpose as designed, many of them were partially relevant. They often captured at least one aspect of benefit, but not the broader functional impact of cBCI use across platforms and real-world settings. One likely reason for this observation is the diversity of potential approaches to cBCI (Vansteensel and Jarosiewicz, 2020). Some forms of cBCI rely on neural activity unrelated to speech or motor behaviors, such as the P300 speller (Rezeika et al., 2018; Farwell and Donchin, 1988) and steady-state visual evoked potential (SSVEP) BCIs (Vialatte et al., 2010; Nakanishi et al., 2018). These types of BCI leverage stereotyped neural responses to visual and attentive stimuli. Some classify between neural functions such as internal speech, arithmetic, and motor imagery, which are so distinct in their activation of cortical circuits that accurate classification is feasible even from noninvasive electrodes (Sorger et al., 2009). Many early BCIs tested with human clinical trial participants used non-speech motor activity to control a computer cursor to type on a virtual keyboard (Pandarinath et al., 2017; Bacher et al., 2015). One novel study used hand-motor neural activity to decode imagined handwriting, obviating the need for the cursor and virtual keyboard (Willett et al., 2021). Another form of cBCI synthesizes audible speech directly from neural activity typically over face (or “speech”) areas of sensorimotor cortices (Akbari et al., 2019; Angrick et al., 2021; Metzger et al., 2023; Moses et al., 2021; Anumanchipalli et al., 2019; Herff et al., 2019). Lastly, there is a class of cBCIs, including ongoing scientific studies which could lead to future cBCIs, which derive their neural inputs from non-motor speech-related areas of the brain, such as the classification of internal speech from the supramarginal gyrus (SMG) (Wandelt et al., 2024) or the observation of semantic encoding in the middle-frontal gyrus (Jamali et al., 2024). Thus, the neural substrates for cBCIs could span the encoding of semantic or conceptual communication intent, speech or non-speech motor signals, vision, and executive functions such as attention. This diversity in cBCI approaches, reflecting the diversity of communication deficits and mechanisms to restore communication, helps explain why existing COAs might have partial relevance, but none were fit for purpose as designed.

### 4.3 Clinically meaningful COAs

KOLs emphasized the need to assess communication at the level of activities required to live independently, i.e., ensuring alignment between the design and intended use of a COA. There are two relevant factors in the design of a COA to meet this need: construct validity and clinical validity. Construct validity requires that the tool addresses the intended measure; clinical validity requires accuracy in measuring the intended state.

Many of the COAs identified as part of the literature review, such as the NASA-TLX and QUEST 2.0 were designed to be applicable to a wide range of tasks and technologies. However, given the complexity of the problem, it is likely that early cBCIs will have narrowly defined intended uses. Should COAs follow suit, specializing to the narrow set of deficits being addressed by a given cBCI, or remain general to overall communication ability? The *concept of interest* (COI), or what the COA is supposed to measure, and *context of use* (COU), or the setting in which it will be used, must be clearly defined (Walton et al., 2015) to ensure construct validity. Together, the COI and COU define the role of the COA in providing a meaningful assessment of the cBCI in a clinical trial.

Next, COAs must be sensitive to clinically meaningful changes for the COI and within the COU and *fit for purpose* (FFP), i.e., there should be sufficient evidence to justify use of the COA as described in the COI and COU. A *clinically meaningful change* is a significant difference in a person’s ability to feel, function, or survive between two assessments. The degree of significance deemed meaningful should be determined by the scorer (patient, observer, or clinician).

Variability in scoring, e.g., due to inter- or intra-rater reliability or test–retest reliability, can confound the clinical validity of a COA even when it has been designed appropriately. COA for cBCI may require specific use to ensure reliability, for example, only in a certain window of functional capacity. Test–retest reliability is susceptible to noise and systemic effects of disease progression. It is essential to formulate a strategy to deal with declining function over time, for example, by comparing continuously to non-BCI best AAC or using control measurements to index into normative data or other statistical approaches.

### 4.4 Frameworks for real-world assessment in cBCIs

KOL interviewees emphasized the need to evaluate communication at the level of real-world activities required to live independently and using communication technologies commonplace in modern society. Here, “independently” refers specifically to communication autonomy, recognizing that many individuals with ALS may still require physical assistance for daily activities. At least one form of COA should address high-level communication capabilities with the cBCI, and these COAs should already account for, or adapt to, the use of technology to perform these tasks.

Traditional activities of daily living (ADL) do not capture the range of deficits in communication associated with ALS, and so other frameworks are required for such an assessment (Nicolelis and Lebedev, 2009; Thompson et al., 2014). Instrumental activities of daily living (iADLs) are activities that support independent living and thus provide a framework for evaluating a cBCI’s utility for communication tasks. Appropriate frameworks may also need to address the use of technology in communication, such as through digital ADLs. *Functional communication* (FC) (Doedens and Meteyard, 2020, 2022; Le and Lui, 2024) views communication more as a means to an end than the end itself, supporting for example the use of technology to address iADLs (Mendoza-Holgado et al., 2024; Melrose et al., 2016; Quamar et al., 2020).

The relevance of digital devices and the need to address accessibility for individuals with impairments were recognized by KOLs and the Clinical Outcomes Review Panel, highlighting the importance of adapting assessment and support tools to align with technological advancements. Overall, integrating BCIs and digital tools into ADLs and iADLs can significantly enhance QOL for individuals, provided these tools are effective, accessible, and aligned with real-world needs and outcomes (Borgnis et al., 2023). For example, if a cBCI cannot be used with the digital tools through which people routinely communicate, its functional benefit may be limited even when laboratory performance is strong. Conversely, effective integration into digital communication environments may substantially enhance quality of life and participation. However, there is an obvious need for clear definitions, consensus on classifications, and robust implementation and measurement frameworks emphasized (Beswick et al., 2021).

### 4.5 Regulatory alignment

Most KOLs, as well as the Clinical Outcomes Review Panel, noted that device performance alone is insufficient to capture the value of a cBCI. Other critical aspects of device experience include usability, ease of learning, and device design (McNicholl et al., 2021; Larsson Ranada and Lidström, 2019), and steep learning curves can lead to long-term dissatisfaction. Both groups also agreed that the progressive nature of ALS, and the complexity and variability of cognitive and motor impairments in ALS, is a challenging context in which to evaluate cBCIs. Ongoing, comprehensive evaluations by neuropsychologists can provide longitudinal data for tracking changes in cognitive function and understanding the impact of BCI systems. Not all cognitive assessments are applicable, but some, like the Edinburgh Cognitive and Behavioral ALS Screen (ECAS) (Siciliano et al., 2017), Attention Network Test (ANT) (Fan et al., 2002), and Flanker Task (Eriksen and Eriksen, 1974), evaluate specific aspects of executive function relevant to ALS patients and the use of cBCI (Carelli et al., 2017). There was, however, a consensus that such evaluations do not lend themselves well to repeated clinical assessment and are better suited for pre-screening than for evaluation of efficacy.

The FDA has defined four categories of COA: Clinician-Reported Outcomes (ClinRO), Observer-Reported Outcomes (ObsRO), Patient-Reported Outcomes (PRO), and Performance Outcomes (PerfO). The literature review performed in this study found only one ClinRO, the Therapeutic Outcome (TOM) measure. One reason for the lack of ClinROs is that there are only a few cases where it would be helpful or necessary to filter outcome scores through a clinician. There were also few ObsROs, where a caregiver or family member might answer questions based on observing the individual with ALS. Caregiver involvement may become increasingly important as the disease progresses and the diagnosed individual requires a greater level of support to use the cBCI. Most of the COAs identified from the literature review were classified as PRO, an apt outcome given the individuality of communication. Most PROs were in the form of QoL assessments, and some of these were specific to the role of communication in QoL. These patient-centric COAs reflect the growing emphasis placed on PROs by the FDA (Matts et al., 2022; Health, Center for Devices and Radiological, 2022).

Recent initiatives by the FDA and its collaborators underscore a growing regulatory focus on developing fit-for-purpose COAs for implanted BCI systems. Notably, the 2023 BCI Society workshop, *Building consensus on clinical outcome assessments for BCI devices*, convened cross-sector stakeholders to address the need for standardized, meaningful COAs tailored to the unique challenges of BCI technologies (Sawyer et al., 2025). Building on this momentum, the 2024 joint FDA/NIH workshop concentrated on advancing COA development for implanted BCIs (Public Workshop – Food and Drug Administration/National Institutes of Health Joint Workshop: Developing Implanted Brain-Computer Interface Clinical Outcome Assessments to Demonstrate Benefit). Additionally, this work was supported by a targeted FDA grant aimed at systematically reviewing COAs for communication BCIs in ALS (ID: RFA-FD-23-030). Parallel efforts, such as the iBCI Collaborative Community’s COA Working Group (Clinical Study Endpoints, 2024) and emerging digital instrumental activities of daily living (diADL) frameworks (Sawyer et al., 2025), are contributing critical perspectives. Collectively, these endeavors highlight a shared commitment to aligning clinical, patient-centric, and regulatory priorities in this evolving framework to develop COAs for cBCIs.

### 4.6 Caregiver burden

Both KOLs and the review panel consistently raised the issue of caregiver satisfaction in the context of COAs for cBCI. Caregivers are often overlooked in assessing the needs of individuals with ALS, but KOLs noted that points of engagement between caregivers and cBCI, such as ease of setup and maintenance, integration into existing routines, and impact on caregiver mental and emotional wellbeing, should be included in clinical assessment. Review panel members noted the importance of assessing the balance between improving the lives of ALS patients and increasing the overall burden on their caregivers. The complex relationship between caregivers and technology necessitates careful consideration of how increased demands or dissatisfaction may manifest. Both formal and informal assessment methods are needed to address practical, emotional, and psychological factors (Eagly and Chaiken, 1993).

Informal and qualitative feedback can reveal practical issues and emotional responses not captured by structured tools, while formal tools provide a quantitative assessment of caregiver stress and patient QOL (Ben Mortenson et al., 2018; Davies et al., 2020). The complementary nature of measuring device performance, QOL, and satisfaction provides a holistic view of the BCI’s impact. While device performance offers objective data, QOL and satisfaction reveal the user’s long-term well-being and personal satisfaction with the device (Borgnis et al., 2023; Garcez et al., 2022). Discrepancies between device performance and QOL/satisfaction highlight the need to address individual user needs and expectations. The distinct characteristics of BCIs, such as invasiveness and specific interaction methods, require tailored evaluation criteria that differ from those used for traditional AAC devices. This tradeoff highlights the importance of developing standardized, well-defined metrics and context-specific evaluations to provide a comprehensive understanding of how these technologies enhance communication (Fry et al., 2022; Maiseli et al., 2023).

### 4.7 Limitations

The purposive sampling approach used for KOL recruitment could introduce selection bias that may limit the diversity of perspectives captured. Furthermore, the explicit exclusion of individuals who disapprove of cBCI as a viable technology for individuals with ALS left critical viewpoints absent from the interview findings.

## 5 Conclusion

This study identified a critical gap in the clinical landscape: no existing COA is fit for purpose to evaluate cBCIs in the ALS patient population. Despite identifying 21 potentially relevant measures across seven COA categories through systematic literature review, none adequately captured the functional impact of cBCI use in the context of ALS. Thematic analysis of KOL interviews reinforced this finding, revealing that existing assessments fail to account for three defining features of the cBCI-in-ALS evaluation problem: the progressive and highly variable nature of communication deficits across disease stages, the technological diversity of cBCI approaches, and the mismatch between laboratory-based performance metrics and real-world communication function. KOLs broadly endorsed communication and ADL-based categories as the most valid assessment targets, while recommending that caregiver and QOL measures serve as secondary, indirect indicators of benefit, and that all COAs be interpreted against the declining functional baseline inherent to ALS progression. The most actionable insight from KOL perspectives and the review panel was the need for future COAs to clearly define their concept of interest and context of use, align with functional communication and iADL frameworks (including digital ADLs), and adopt longitudinal measurement strategies that account for disease progression. These findings carry direct implications for clinical trial design and regulatory evaluation: a comprehensive, cBCI-specific COA battery, anchored in real-world communication function and validated for the ALS population, is needed before meaningful efficacy claims can be substantiated. The present work provides an evidence-based foundation for that development effort, aligned with parallel initiatives from the FDA, BCI Society, and emerging community working groups.

## Data Availability

The raw data supporting the conclusions of this article will be made available by the authors, without undue reservation.
