# Supplementary material for: Clinical evaluation of communication brain computer interfaces in amyotrophic lateral sclerosis: a landscape analysis
Source: Front Hum Neurosci. 2026 May 22;20:1771146. doi: 10.3389/fnhum.2026.1771146 (PMC13236962; doi:10.3389/fnhum.2026.1771146)
Supplement: Supplementary file 1 [file Data_Sheet_1.PDF]

INTERVIEW SCRIPT—KEY OPINION LEADER INTERVIEWS

[Introduction]

Interviewer: Today, I would like to ask you some questions about Clinical Outcome Assessments (COAs) to evaluate communication in patients with ALS. The FDA defines a COA as "a measure that describes or reflects how a patient feels, functions, or survives." For the purposes of this interview, we are considering COAs that could be used to evaluate how well an intervention, like a brain-computer interface (BCI), improves communication for an individual with ALS. Developing a standardized COA is critical for future clinical trials that will attempt to measure the value of BCIs for these individuals.

Based on a systematic literature review of publications studying BCIs and other technologies intended to improve communication for persons with ALS, we compiled a list of relevant tools and grouped them into several different categories. Over the course of the interview, I'll ask for your opinions and feedback about assessments in each of these categories.

Are you comfortable proceeding?

Interviewee: (Response)

- Yes → proceed.
- No → record study exit.

[Beginning Of Interview]

Interviewer: Great. Let's get started. To begin, how would you measure the clinical benefit of an intervention intended to improve communications for a person with ALS?

Interviewee: (Response)

Interviewer: (*Shows table of COA categories with descriptions; duplicate of Table 3 from Research Protocol—Literature Review and Analysis)*

Assessment Categories	Description
Activities of daily living (ADL)	Addressing daily functional needs
Assistive technology (AT) performance	Capability of assistive technologies to meet user needs
Brain-computer interface (BCI) performance	Assessment specific to BCI use
Caregiver	Health and wellbeing of caregivers, and ability of caregiver to perform duties
Cognitive and Neurological	Neurological assessment of cognitive ability
Communication	Clinical assessment of ability to communicate
Patient Satisfaction and Quality of life (QoL)	Self-reported assessment of satisfaction around use of assistive technology and an individual's life satisfaction

Table 3. Categories of assessment tools.

Interviewer: Which of these categories are relevant to your domain of expertise?

Interviewee: (Response)

Interviewer: In your opinion, are these the right categories? Would you add, remove, or modify any categories?

Interviewee: (Response)

- If interviewee suggests any change(s): “Can you elaborate why [*category*] should be [*removed*/*modified*/*added*]?”
- If interviewee has indicated any disagreement with existing categories, explain, “Thank you for this feedback. For the purposes of this interview, to ensure we record responses to a common set of questions, we will still ask for your opinions about each of these predetermined categories. Please continue to provide critical feedback during those sections of the interview. Afterward, however, we will follow up on your feedback with additional questions.”

[ADL Assessments]

Interviewer: I will start with the ADL category of assessments. You may already be familiar with the terminology activity of daily living (ADL) or instrumental activities of daily living (IADL), however, to make sure we are working from the same definition, we define these terms in the following way. ADLs are basic self-care tasks and include walking, feeding, bathing, etc. IADLs are self-care tasks that require more complex thinking skills, and include managing finances, managing transportation, shopping and meal preparation, etc.

In your opinion, would a tool designed to measure ADL or IADL performance be a valid tool for measuring the clinical benefit of a device that enables communication?

Interviewee: (Response)

- Yes → proceed.
- No → “Can you please elaborate why you do not believe ADL/IADL assessments would be valid for evaluating communication?”

Interviewer: Even if they are not on this list, which ADL/IADL assessment tools are best suited to evaluate the performance of an assistive device for communication?

Interviewee: (Response)

Interviewer: Does enabling a patient to communicate affect IADLs?

Interviewee: (Response)

- Yes → “How is communication typically measured in the context of IADLs?”
- No → proceed.

Interviewer: Please review this list of assessment tools we have identified so far for ADLs and IADLs.

(*Shows list of COAs in this category)*

Interviewer: Are there any tools on this list you would consider irrelevant, invalid, incomplete, or otherwise unsuited for evaluating communication in ADLs or IADLs?

Interviewee: (Response)

Interviewer: Is there a tool you do not see on this list that could be used to evaluate communication in ADLs or IADLs?

Interviewee: (Response)

Interviewer: We propose creating a new classification of ADLs, called Digital ADLs, that encompasses interactions with computers, phones, and other devices that are required for ADLs or IADLs. What do you think about this proposal?

Interviewee: (Response)

- Positive → "If you were designing a tool to measure performance in Digital ADLs/IADLs, what would you measure?"
- Negative → "How would you capture interactions with compute devices necessary for ADLs and IADLs?"

Interviewee: (Response)

[Assistive Technology Performance]

Interviewer: I will now ask questions about the Assistive Technology Performance category. In this section, we will use the terms assistive technology (AT) and augmentative and alternative communication (AAC) to refer to interventions designed to assist disabled persons, specifically around communication in the case of AAC. In your opinion, is this a valid category for measuring the clinical benefit of a device that enables communication?

Interviewee: (Response)

Interviewer: Is it acceptable to use a tool validated for Assistive Technology or AAC to assess a BCI?

Interviewee: (Response)

Interviewer: In your experience, what are the most relevant and appropriate measures for evaluating the performance of assistive technology, specifically BCI systems, for communication?

Interviewee: (Response)

Interviewer: What are some key factors in performance or device usage that should be captured to properly assess assistive technologies for communication?

Interviewee: (Response)

Interviewer: Are there any such factors unique to brain-computer interfaces?

Interviewee: (Response)

Interviewer: In your experience, is device performance a strong predictor of patient satisfaction?

Interviewee: (Response)

- Yes or No → "Can you please elaborate why?"

Interviewer: Please review this list of assessment tools we have identified so far for Assistive Technology for communication.

(*Shows list of COAs in this category)*

Interviewer: Are there any tools on this list you would consider irrelevant, invalid, incomplete, or otherwise unsuited for evaluating Assistive Technology for communication?

Interviewee: (Response)

Interviewer: Is there a tool you do not see on this list that could be used to evaluate assistive technology performance?

Interviewee: (Response)

[Brain Computer Interface Performance and Usability Assessments]

Interviewer: I will now ask questions about the BCI Performance and Usability Assessments category. In your opinion, is this a valid category for measuring the clinical benefit of a device that enables communication?

Interviewee: (Response)

Interviewer: Should BCIs be evaluated differently than AT/AAC in terms of ability to improve communication?

Interviewee: (Response)

- Yes → "How should these differences be addressed?"
- No → proceed.

Interviewer: Please review this list of assessment tools we have identified so far for BCI Performance and Usability.

(*Shows list of COAs in this category)*

Interviewer: Are there any tools on this list you would consider irrelevant, invalid, incomplete, or otherwise unsuited for evaluating the performance and usability of brain computer interfaces?

Interviewee: (Response)

Interviewer: Is there a tool you do not see on this list that could be used to evaluate the performance and usability of brain computer interfaces?

Interviewee: (Response)

[Caregiver Assessments]

Interviewer: I will now ask questions about the Caregiver Assessments category. In your opinion, is this a valid category for measuring the clinical benefit of a device that enables communication?

Interviewee: (Response)

Interviewer: Do you think caregiver assessments should be included when measuring the utility of an intervention designed to improve communication for individuals with ALS?

Interviewee: (Response)

Interviewer: What tools would you use to assess caregiver impact?

Interviewee: (Response)

Interviewer: Please review this list of tools we have identified so far for caregiver assessment.

(*Shows list of COAs in this category)*

Interviewer: Are there any tools on this list you would consider irrelevant, invalid, incomplete, or otherwise unsuited for evaluating caregiver impact?

Interviewee: (Response)

Interviewer: Is there a tool you do not see on this list that could be used to evaluate caregiver impact?

Interviewee: (Response)

[Cognitive and Neurological Assessments]

Interviewer: I will now ask questions about the Cognitive and Neurological Assessments category. In your opinion, is this a valid category for measuring the clinical benefit of a device that enables communication?

Interviewee: (Response)

Interviewer: What role does cognitive load play in determining the utility of an AT/AAC system for an individual?

Interviewee: (Response)

Interviewer: Should cognitive load be captured when assessing an AT or AAC device?

Interviewee: (Response)

Interviewer: In your experience, how do patients or users assess cognitive burden when evaluating a new AT/AAC system, and/or when discussing their existing AT/AAC systems?

Interviewee: (Response)

Interviewer: How should a COA account for individual differences in cognitive and neurological function when designing a measure to evaluate BCI systems for communication?

Interviewee: (Response)

Interviewer: Are there any cognitive or neurological aspects unique to paralyzed patients that may affect their ability to use a BCI system for communication effectively?

Interviewee: (Response)

Interviewer: Are there specific cognitive or neurological assessment tools you would recommend for evaluating the impact of a BCI system on communication in paralyzed patients?

Interviewee: (Response)

Interviewer: Are there any pre-screening assessments that you are aware of that could be used to assess cognitive ability to use a BCI?

Interviewee: (Response)

Interviewer: Please review this list of tools we have identified so far for cognitive and neurological assessment.

(*Shows list of COAs in this category)*

Interviewer: Are there any tools on this list you would consider irrelevant, invalid, incomplete, or otherwise unsuited for cognitive and neurological assessment in the ALS population?

Interviewee: (Response)

Interviewer: Is there a tool you do not see on this list that could be used to assess cognitive and neurological function in ALS?

Interviewee: (Response)

[Communication Outcomes]

Interviewer: I will now ask questions about the Communications Outcomes category. In your opinion, is this a valid category for measuring the clinical benefit of a device that enables communication?

Interviewee: (Response)

Interviewer: If you were evaluating a patient to determine whether to recommend a BCI system, how would you screen for communication needs?

Interviewee: (Response)

Interviewer: Are you aware of any tools or classification frameworks to evaluate benefit and risk in communication? For example, how would you balance communication error rate versus the need to provide informed consent for a medical procedure?

Interviewee: (Response)

Interviewer: Do you feel that assessing Communication Outcomes is a valid approach for measuring the clinical benefit of a BCI device that enables communication?

Interviewee: (Response)

Interviewer: In your experience, what are the key factors that contribute to successful communication outcomes for ALS patients using existing assistive technologies?

Interviewee: (Response)

- **Follow-up:** “Would these factors change if you were considering communication through a BCI?”

Interviewer: Are there technical metrics specific to BCI that should be assessed as part of a COA?

Interviewee: (Response)

- If yes, ask to elaborate.

Interviewer: How should BCI systems be measured against existing AT/AAC interventions in the context of communication for individuals with ALS?

Interviewee: (Response)

Interviewer: Please review this list of assessment tools we have identified so far for Communication Outcomes.

(*Shows list of COAs in this category)*

Interviewer: Are there any tools on this list you would consider irrelevant, invalid, incomplete, or otherwise unsuited for evaluating communication outcomes?

Interviewee: (Response)

Interviewer: Is there a tool you do not see on this list that could be used to evaluate communication outcomes in ALS patients?

Interviewee: (Response)

[Patient Satisfaction and Quality of Life Assessments]

Interviewer: I will now ask questions about the Patient Satisfaction and QoL Assessments category. Do you feel this is a valid category for measuring clinical benefit of a device that enables communication?

Interviewee: (Response)

Interviewer: Interviewer: Is it acceptable to use a tool validated for QoL and satisfaction to assess AAC interventions like BCI?

Interviewee: (Response)

Interviewer: What criteria would you use to select a QoL and satisfaction assessment tool for evaluating the impact of assistive technology in persons with ALS?

Interviewee: (Response)

Interviewer: In your opinion, are QoL and satisfaction useful to measure independently of, and/or in addition to, device performance?

Interviewee: (Response)

Interviewee: (Response)

Interviewer: Please review this list of assessment tools we have identified so far for Patient Satisfaction and Quality of Life.

(*Shows list of COAs in this category)*

Interviewer: Are there any tools on this list you would consider irrelevant, invalid, incomplete, or otherwise unsuited for evaluating patient satisfaction and QoL?

Interviewee: (Response)

Interviewer: Is there a tool you do not see on this list that could be used to evaluate ALS patient satisfaction and quality of life?

Interviewee: (Response)

[OPTIONAL: Template to Follow Up on Suggested Categories]

Interviewer: I will now ask questions about the category [*category name*] you proposed at the beginning of the interview. Can you elaborate why you feel this is a valid category for measuring clinical benefit of a device that enables communication?

Interviewee: (Response)

Interviewer: What existing assessment tools, if any, you would place in this category?

Interviewee: (Response)

Interviewer: Would the use of these tools change at all if they were used to evaluate a BCI for communication in ALS patients instead of their intended use?

Interviewee: (Response)

- Yes → "How should these differences be addressed?"
- No → proceed.

[Closing of Interview]

Interviewer: We have finished all the questions I have for you today. Is there anything else you would like to add that would be relevant to this topic?

Interviewee: (Response)

[Wrap-Up]

Interviewer: I appreciate your time and valuable insights. Thank you.

[End of Interview]
